# Impact of Influenza on Outpatient Visits, Hospitalizations, and Deaths by Using a Time Series Poisson Generalized Additive Model

**DOI:** 10.1371/journal.pone.0149468

**Published:** 2016-02-19

**Authors:** Ru-ning Guo, Hui-zhen Zheng, Chun-quan Ou, Li-qun Huang, Yong Zhou, Xin Zhang, Can-kun Liang, Jin-yan Lin, Hao-jie Zhong, Tie Song, Hui-ming Luo

**Affiliations:** 1 Public Health Emergency management office, Center for Disease Control and Prevention of Guangdong Province, Guangzhou, China; 2 Institute of Immunization Programs, Center for Disease Control and Prevention of Guangdong Province, Guangzhou, China; 3 Department of Biostatistics, School of Public Health, Southern Medical University, Guangzhou, China; 4 Zhuhai Municipal Center for Disease Control and Prevention, Zhuhai, China; 5 Institute of Pathogenic Microorganisms, Center for Disease Control and Prevention of Guangdong Province, Guangzhou, China; 6 Center for Disease Control and Prevention of Guangdong Province, Guangzhou, China; 7 Institute of Infectious Disease Prevention and Control, Center for Disease Control and Prevention of Guangdong Province, Guangzhou, China; 8 Center for Disease Control and prevention, Beijing, China; National Center for Immunization and Respiratory Diseases, UNITED STATES

## Abstract

**Background:**

The disease burden associated with influenza in developing tropical and subtropical countries is poorly understood owing to the lack of a comprehensive disease surveillance system and information-exchange mechanisms. The impact of influenza on outpatient visits, hospital admissions, and deaths has not been fully demonstrated to date in south China.

**Methods:**

A time series Poisson generalized additive model was used to quantitatively assess influenza-like illness (ILI) and influenza disease burden by using influenza surveillance data in Zhuhai City from 2007 to 2009, combined with the outpatient, inpatient, and respiratory disease mortality data of the same period.

**Results:**

The influenza activity in Zhuhai City demonstrated a typical subtropical seasonal pattern; however, each influenza virus subtype showed a specific transmission variation. The weekly ILI case number and virus isolation rate had a very close positive correlation (r = 0.774, P < 0.0001). The impact of ILI and influenza on weekly outpatient visits was statistically significant (P < 0.05). We determined that 10.7% of outpatient visits were associated with ILI and 1.88% were associated with influenza. ILI also had a significant influence on the hospitalization rates (P < 0.05), but mainly in populations <25 years of age. No statistically significant effect of influenza on hospital admissions was found (P > 0.05). The impact of ILI on chronic obstructive pulmonary disease (COPD) was most significant (P < 0.05), with 33.1% of COPD-related deaths being attributable to ILI. The impact of influenza on the mortality rate requires further evaluation.

**Conclusions:**

ILI is a feasible indicator of influenza activity. Both ILI and influenza have a large impact on outpatient visits. Although ILI affects the number of hospital admissions and deaths, we found no consistent influence of influenza, which requires further assessment.

## Introduction

A large number of evidence shows that influenza can lead to increased mortality rates due to heart and lung disease, which can cause great epidemiological and economic burdens to the community [1–11]. The average annual incidence of influenza-associated hospitalizations is particularly high in children <5 years of age [12, 13], greatly promoting the development of vaccination strategies. The findings of a population-based study indicated that the influenza-associated disease burden on young children were mainly derived from outpatients, involved a high attendance rate, common complications, and immeasurable missed work/absenteeism, followed by influenza-associated hospitalizations, and death-related disease burden caused by influenza was comparatively low [14]. Studies to date have been primarily performed in developed temperate countries, as well as in a few high-income districts in tropical and subtropical countries [2, 4, 10]. However, basic data on influenza burden are lacking for most tropical and subtropical countries owing to lagging health-care facilities, monitoring, and information collection systems [15].

The epidemiological features of influenza vary greatly across regions and climates. In the temperate regions of Europe and America, the influenza activity has a distinct seasonal pattern, with a winter epidemic peak and almost no activity during the other seasons. In subtropical areas such as Guangdong Province in south China, however, influenza activity occurs throughout the year, presenting a vague seasonality that makes it more difficult to use mathematical models that are frequently adopted in temperate areas [13, 16]. Studies on the influenza burden and the findings in temperate regions cannot be directly applied to (sub) tropical areas because of local features and population-specific attributes. In recent years, research on influenza-related hospitalizations conducted in central China has shown that a seasonal influenza epidemic causes a substantial number of hospitalizations [17], with an estimated 115–142 patients with severe acute respiratory infections per 100 000 hospitalizations attributed to influenza. However, the degree of impact of influenza in subtropical southern China has yet to be reported.

Our study group helped evaluate the large influence of influenza and influenza-like illness (ILI) on outpatient visits in view of morbidity and direct economic burden [18, 19]; however, the impact of influenza on hospitalizations and deaths has never been examined in southern China, an area once considered by experts as the “epicenter of influenza pandemic” [20] and the origin or high-risk location of multiple emerging infectious diseases such as severe acute respiratory syndrome [21] and human infection with avian influenza H7N9 [22].

Located in a subtropical area of south China, Zhuhai City in Guangdong Province has a well-established community health and influenza surveillance system that provides a good platform for the study of influenza disease burden. In this study, the time series Poisson generalized additive model (GAM) was used to quantitatively assess the disease burden of influenza and ILI, by using the influenza surveillance data in Zhuhai City from 2007 to 2009 combined with outpatient, inpatient, and respiratory disease mortality data from the same period.

## Materials and Methods

### Study community and data sources

With an area of 1701 km^2^ and a population of 156.02 million (2010 census), Zhuhai City, the smallest city in Guangdong Province, was selected as the research community. The city has a moderate level of economic development and population density within the province, as well as a well-established community health and influenza surveillance system. It has a typical subtropical monsoon climate (annual average temperature, 22.3°C; annual rainfall, 1770–2300 mm).

Zhuhai City has a far-reaching influenza surveillance network. The outpatient and ILI surveillance data of this study were taken from the monitoring networks of 28 medical institutions (covering most medical services). ILI is defined as an illness with a protrusion fever (body temperature ≥38°C), cough, sore throat, or other respiratory symptoms. The ILI visit percentage (ILI %) refers to the proportion of hospital visits for ILI within a certain period to the total number of outpatient visits for the same period, and is used as an indicator of the degree of influenza epidemic.

The influenza surveillance data were derived from 14 of 28 medical institutions. Influenza cases were those involving patients whose throat swabs were isolated and who were positive for the influenza virus in the cell culture assay (Madin-Darby canine kidney cells). The rate of influenza virus isolation refers to the proportion of influenza virus–positive specimens to the total number of detected specimens. Weekly ILI surveillance and pathogen monitoring data were analyzed. Inpatient data were collected monthly from 18 hospitals in the city where inpatient care was provided during 2004–2009. However, only three hospitals provided inpatient data from 2007 to 2009 on a weekly basis, and thus we counted the weekly hospital admissions and established our model analysis on the basis of these data.

The citywide death cases were collected during 2006–2008. Death certificates were issued by hospitals, community health centers, or rural clinics and entered into the Death Registration Information System. The causes of death were based on the death classification criteria in the World Health Organization’s (WHO) International Classification of Diseases (10th edition). The major causes of deaths related to respiratory disease (J00–J99) in Zhuhai City include pneumonia (J9–J18), cardiopulmonary diseases (J26–J28), chronic obstructive pulmonary disease (COPD) (J40–J47), and others.

### Statistical analysis

SPSS 13.0 statistical software and R language version 2.3 were used for the data analysis. The Pearson correlation method was adopted for the analysis of the correlation among the number of weekly ILI, outpatient visits, and influenza cases (virus isolation rate). The GAM was used to fit the regression relation among weekly outpatient visits, hospital admissions, and respiratory deaths, as well as to assess the impact of ILI and influenza on these indicators. The model analysis method was as follows (with respiratory deaths as an example).

The GAM was adopted to fit the regression model for weekly respiratory deaths. By using the average weekly temperature and relative humidity with the cubic spline smoothing function to control for the confounding influence of potential meteorological factors, an additional time (*t* = 1, 2, …, 105) smoothing function was constructed to filter out the long-term death trends, seasonality, and other confounding factors that failed measurements. The established core models were as follows [3, 5]:

$$\begin{array}{l} Y_{t}\sim Poisson(\mu_{t})\\ E(Y_{t})=\mu_{t}\;Var(Y_{t})=\phi \mu_{t}\\ \text{log}E(Y_{t})=\alpha +f(t)+q(z_{t}) \end{array}$$ (1)

where *Y*_*t*_ is the dependent variable, the number of deaths per week; *μ*_*t*_ is the expected mean,*Y*_*t*_; *ϕ* is the over-dispersion parameter (given *ϕ* is equal to 1, the outcome perfectly follows Poisson distribution. *ϕ* more than 1 means over-dispersion and the variance is modified by multiplying *ϕ*); α is the intercept; *f* is a smooth function with respect to the time variable *t*; *z*_*t*_ is a time-varying variable that can be monitored, for example, meteorological factors such as temperature and relative humidity; and *q* is a smooth function of *z*_*t*_, for which the cubic spline smoothing function is adopted. Each smooth function has a parameter (e.g., degrees of freedom) that needed to be adjusted to choose the best model. The white noise of residual distribution and non-statistically significant autocorrelation of the partial autocorrelation function was required for the model. Only conditions that met the above criteria could be considered sufficient for controlling the confounding factors. If multiple models met the above criteria, then we chose the smallest function value as the optimal model by using Akaike information criteria. If the residual partial autocorrelation plot always showed significant autocorrelations regardless of model parameter adjustment, the autoregressive term was introduced to establish the autocorrelation regression model for correction.

The study factor *X* (i.e., influenza variable) was introduced into the established core model one at a time as follows:

$$\text{log}E(Y_{t})=\alpha +f(t)+q(z_{t})+\beta X$$ (2)

where *X* is an influenza-related variable and *β* is the regression coefficient. The weekly ILI% and weekly influenza virus isolation rate were selected as indicators reflecting influenza activity, to assess the impact of ILI and influenza on mortality.

The percentage of influenza-associated excess deaths over the total number of deaths was used to reflect the impact of influenza on mortality. Eq 2 was used to calculate the expected weekly actual number of deaths ($E_{X_{i}=x_{i}}$) and the theoretical number of deaths each week ($E_{X_{i}=0}$) when there is no influenza activity (*X* = 0). After dividing the sum of the weekly difference of $E_{X_{i}=x_{i}}$ minus $E_{X_{i}=0}$ by the sum of $E_{X_{i}=x_{i}}$, the excess ratio (*D*_*flu*_) caused by influenza is obtained:

$$D_{flu}=\sum_{i=1}^{n}(E_{X_{i}=x_{1}}-E_{X_{i}=0})/\sum_{i=1}^{n}E_{X_{i}=x_{1}}*100\%$$

The same model analysis method (GAM) was used to fit the correlation between influenza activity and weekly hospitalizations and outpatient visits. Excess hospital admissions and the total outpatient number were also obtained to assess the impact of ILI and influenza on local hospitalizations and outpatient visits.

### Ethics statement

This study was conducted through a collection of historical data of routine influenza surveillance, combined with outpatient, inpatient, and death data from local medical institutions, to construct a statistical model for assessing the ILI and influenza burden on the local residents. Collecting throat swabs from patients with ILI has become a routine diagnostic procedure and thus has a normal public health response. The process of throat swab specimen collection has an extremely small impact on subjects and a risk no greater than that of routine physical or psychological examinations. The study complied with the Helsinki Declaration requirements for the use of human subjects, and received approval from the ethics committee of the Centers for Disease Control and Prevention of Guangdong Province. Oral informed consent was obtained from each participant before the examination of their throat swab after a thorough explanation of the study’s purposes and risks. All data were analyzed anonymously, and any patient identifier was removed in order to maintain patient confidentiality.

## Results

### Influenza activity

The total annual number of outpatients in Zhuhai City in 2007–2009 showed an obvious increasing trend, with an annual average growth rate of 22%. The number of ILI cases fluctuated between years, with a weekly average ILI% of 4.6%, 3.3%, and 4.1%, respectively, whereas the weekly average influenza virus isolation rates were 5.29%, 6.86%, and 26.80%, respectively (Table 1).

**Table 1:** Influenza-like illness (ILI) cases, outpatient visits, and influenza virus positive isolation rate by week (2007–2009, Zhuhai City, China).

Year		Mean	Standard deviation	Median	Min	Max	P25*	P75*
2007	No. ILI cases	928	322	855	549	2281	704	1051
	No. outpatients	19699	2311	19966	11602	24171	18522	21001
	ILI/outpatient (%)	4.6	1.2	4.4	2.9	9.4	3.9	5.3
	No. specimens	18.89	9.9	18	0	40	13	26
	No. positive	1.19	2.1	0	0	8	0	2
	Isolation rate (%)	5.29	9.3	0	0	44.44	0	7.8
2008	No. ILI cases	795	312	725	230	1870	617	1010
	No. outpatients	24353	4773	24673	10992	33485	21278	28097
	ILI/outpatient (%)	3.3	1.1	3	1.7	6.7	2.4	4
	No. specimens	28.56	13.5	27	0	58	20	38
	No. positive	2.6	4.1	1	0	15	0	3
	Isolation rate (%)	6.86	9.3	2.7	0	31.91	0	10.3
2009	No. ILI cases	1430	708	1478	453	3119	737	1965
	No. outpatients	33575	7682	31038	16778	52011	28873	40760
	ILI/outpatient (%)	4.1	1.5	4.2	2.0	7.0	2.6	5.3
	No. specimens	41.23	14.9	43	0	69	31.25	51.8
	No. positive	11.62	10.6	9	0	44	3	19
	Isolation rate (%)	26.8	22.6	21.7	0	79.1	6.5	46.3
Total	No. ILI cases	1050.25	2.3	871	230	3119	680	1236.5
	No. outpatients	25836	7875	23575	10992	52011	20225	30013
	ILI/outpatient (%)	4.0	1.4	4	1.7	9.4	2.8	5
	No. specimens	29.49	15.815	28	0	69	18	40
	No. positive	5.11	8.054	1	0	44	0	7.5
	Isolation rate (%)	12.93	17.88	5.79	0	79.07	0	20

Note:

* P25 and P75 represent 25% and 75% percentiles, respectively.

**Fig 1:**
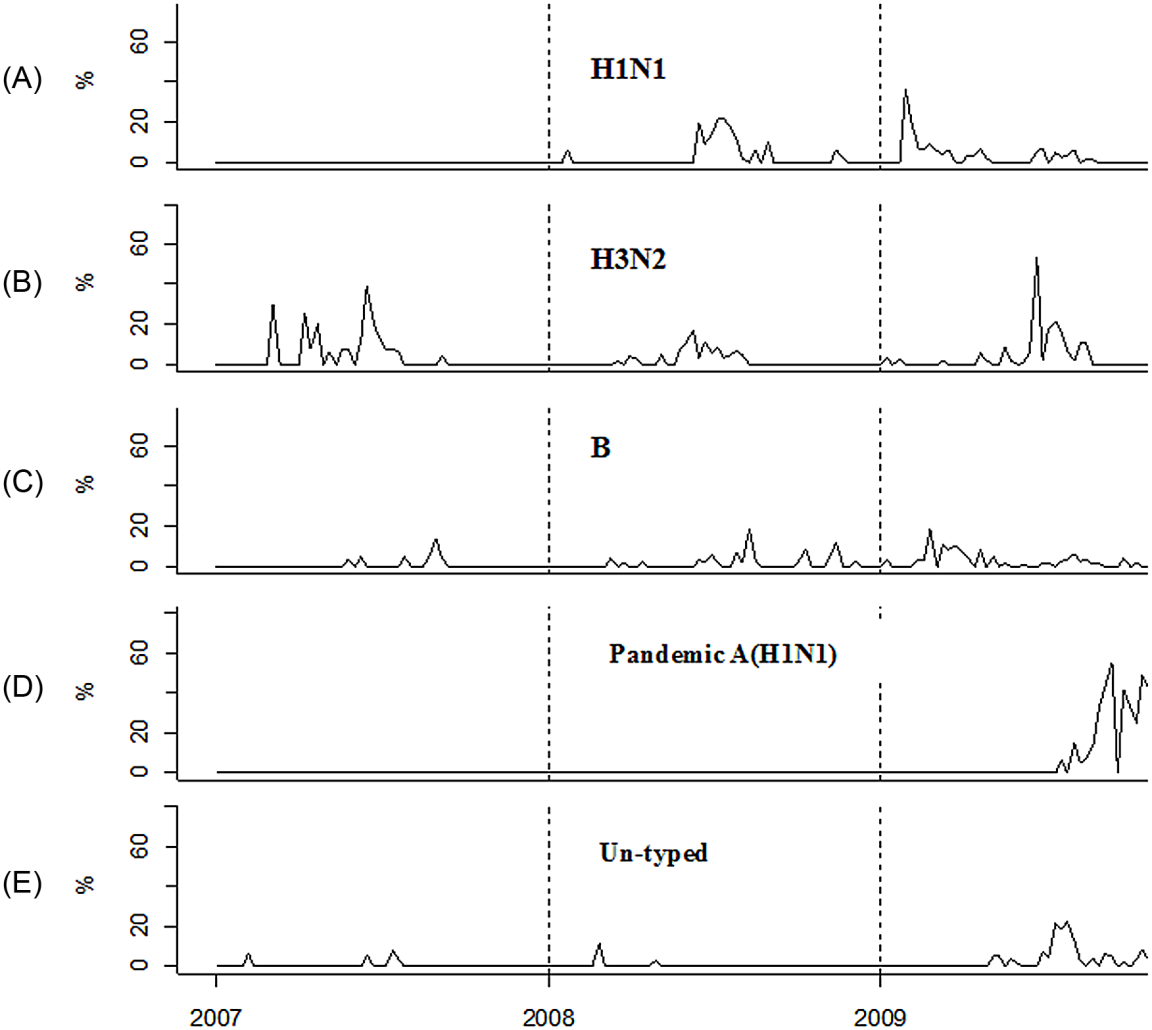
Seasonal transmission pattern of influenza viruses, 2007–2009. Three years of influenza pathogen surveillance data were collected in Zhuhai City, Guangdong Province, China. Specific patterns of seasonal transmission were observed in seasonal influenza A (H1N1, H3N2), influenza B, pandemic influenza A (H1N1), and untyped influenza viruses. The Y-axis refers to the isolation rate of influenza viruses, which is the proportion of the number of influenza virus–positive specimens to the total number of specimens detected.

A total of 802 influenza viruses were isolated during 2007–2009; of them, 257 were seasonal influenza A (H1N1 118, H3N2 149), 95 were seasonal influenza B, 358 were pandemic influenza A (H1N1), and 82 were untyped viruses. Each subtype presented a certain trend of seasonal variation (Fig 1). (i) The prevalence of seasonal influenza A (H1N1) varied by year, with no isolation in 2007, the emergence of a peak in the summer of 2008, and the highest winter peak (January–February) in 2009 (accounting for >33% of the total isolates). (ii) The prevalence of seasonal influenza A (H3N2) was characterized by a significant summertime seasonal peak and a relatively low activity in the spring and winter. (iii) Seasonal influenza B virus presented a summertime peak in 2007 and was active throughout 2008–2009. (4) Pandemic influenza A (H1N1) first appeared in mid-2009, showed a rapidly increasing trend, and in September began to dominate a variety of influenza viruses (80% of the total isolates in November), and then began to decline. (v) Finally, untyped influenza viruses showed low activity during 2007–2008, had increased epidemic activity in 2009 with a significant summertime peak, and reemerged in wintertime.

### Correlation between ILI and influenza

**Fig 2:**
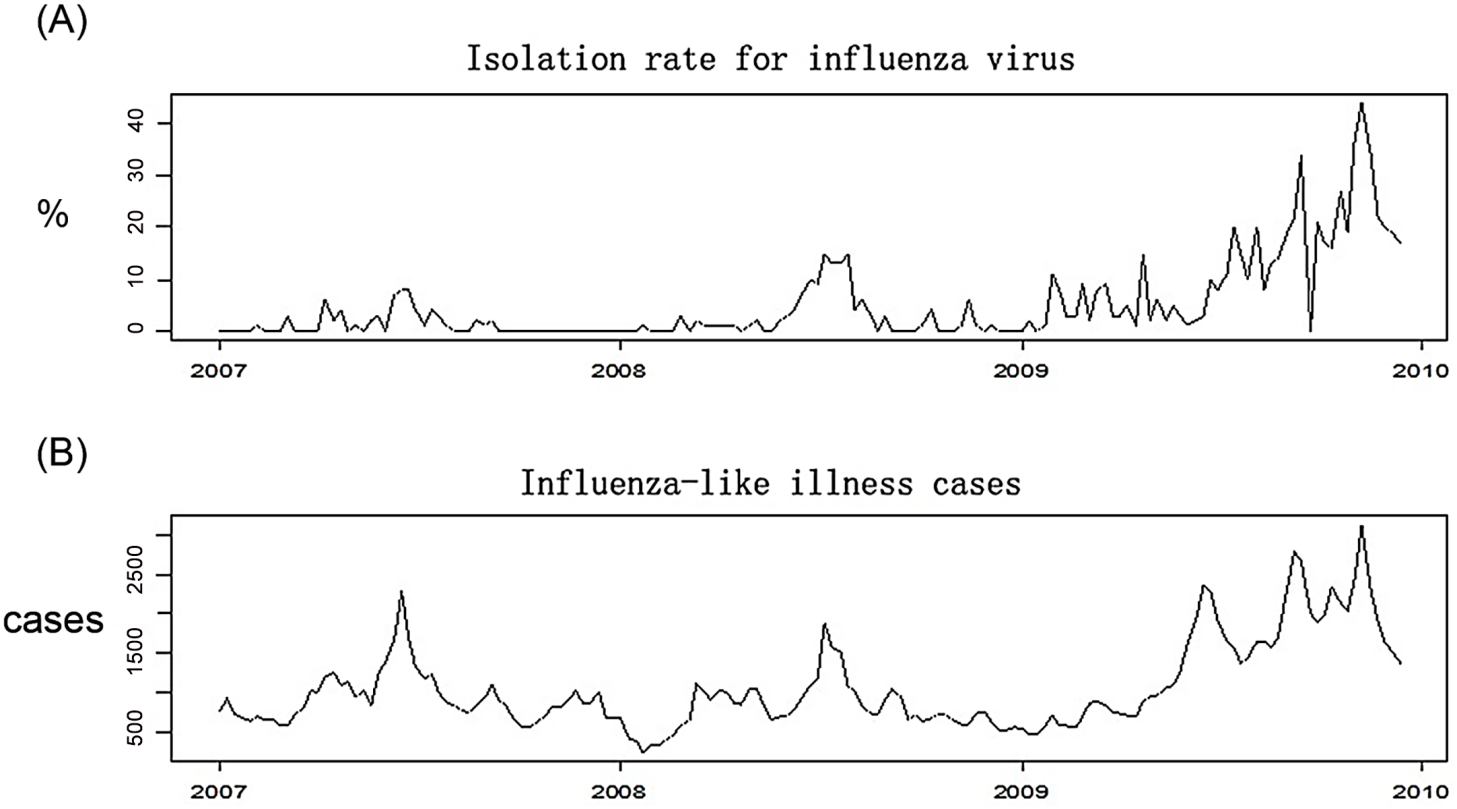
Seasonal pattern of influenza-like illness (ILI) and influenza by week, 2007–2009, Zhuhai City. Three years of ILI and influenza virus surveillance data were collected in Zhuhai City, Guangdong Province, China. A, the Y-axis shows the isolation rate (%) of influenza virus strains per week, which is the proportion of the number of influenza virus–positive specimens to the total number of specimens detected. B, the Y-axis shows the number of patients with ILI per week.

The Pearson correlation analysis of weekly ILI case numbers and a positive isolation rate during 2007–2009 indicated that these two variables had a very close positive correlation (r = 0.774, P < 0.0001). The time series diagram in Fig 2 shows the consistent increasing trend of the weekly ILI case number and the positive isolation rate.

### Model analysis results

Table 2 shows the statistical results of each indicator by week in the model.

**Table 2:** Weekly statistical results of the main indicators in the time series Poisson generalized additive model^#^.

Variables	Mean	Standard deviation	Median	Min	Max	P_25_	P_75_
No. ILI cases	1050.25	2.30	871	230	3119	680	1236.5
No. outpatients*	25836	7875	23575	10992	52011	20225	30013
ILI/outpatient (%)**	4.01	1.40	4.00	1.70	9.44	2.82	5.00
Isolation rate (%)**	12.93	17.88	5.77	0	79.07	0	20
No. hospitalizations*	337	50	342	132	436	312	369
Mean temperature(°C)	22.78	5.38	24.00	6.20	32.00	19.00	27.40
Relative humidity (%)	76	13	78	32	95	69	86

Note: ILI% is the percentage of influenza-like illness (ILI) cases out of the total outpatient visits. The positive rate is the proportion of the number of influenza virus–positive specimens to the total specimens detected. Mean temperature and relative humidity were used in the model for controlling for the potential confounding influence of meteorological factors.

* Dependent variables in the model;

** independent variable, which stands as influenza proxy.

^#^, estimates that are aggregated results from multiple separate models, which means influenza proxy (**) are included in the model one at a time.

### Impact of ILI and influenza on outpatient visits

The impact of ILI on the numbers of weekly outpatient visits during 2007–2009 was statistically significant (P < 0.05). Approximately 10.7% of outpatient visits were ILI related; that is, an average of 2764 patient visits per week were due to ILI. The weekly influenza virus positive isolation rate also had a statistically significant influence on the outpatient visits per week (P < 0.05). Approximately 1.88% of all outpatient visits were due to influenza, with an average of 487 influenza-associated outpatient cases per week; that is, if there was no influenza activity, the outpatient visits declined by 1.88% (Table 3).

**Table 3:** Impact of influenza-like illness and influenza on outpatient visits.

		No. outpatient	Excess outpatient visits	Excess percentage (%)	P value
2007~2009	ILI%	4056320	433947	10.7	0.049
	Positive rate	4056320	76417	1.88	0.042
2009	ILI%	1745899	296312	16.97	0.012
	Positive rate	1745899	36496	2.09	0.106
2008	ILI%	1266356	106526	8.41	0.179
	Positive rate	1266356	23145	1.83	0.223
2007	ILI%	1044065	104027	9.96	0.03
	Positive rate	1044065	11103	1.06	0.074

Note: ILI% is the percentage of the number of cases of influenza-like illness out of the total outpatient visits. The positive rate is the proportion of the number of influenza virus–positive specimens to the total number of detected specimens.

### Impact of ILI and influenza on hospitalizations

ILI had a significant effect on the hospital admission rate (P < 0.05). A total of 3573 cases of hospital admissions were ILI-related during 2007–2009, accounting for 6.76% of all hospital admissions; that is, if there was no occurrence of ILI, the number of hospital admissions would be decreased by about 7%. The analysis of the impact of ILI on hospitalizations by age groups revealed that ILI mainly affected persons <25 years of age but had no significant impact on those aged >25 years. There were 470 influenza-associated hospitalizations, accounting for about 1% of all hospital admissions; however, the effect was not statistically significant (P > 0.05) (Table 4).

**Table 4:** Impact of influenza-like illness and influenza on hospitalizations.

		No. total hospitalizations	No. excess hospitalizations	Percentage of excess hospitalizations (%)	P value
Total population	ILI%*	52908	3573	6.76	0.037
	Positive rate*	52908	470	0.89	0.105
0–4 years	ILI%	2036	382	18.75	0.02
	Positive rate	2036	-	-	0.288
5–14 years	ILI%	1558	98	6.34	0.487
	Positive rate	1558	9	0.56	0.062
15–24 years	ILI%	8252	1197	14.5	0.003
	Positive rate	8252	213	2.59	0.049
25–59 years	ILI%	29672	1452	4.89	0.335
	Positive rate	29672	220	0.28	0.284
≥60 years	ILI%	11390	193	1.7	0.798
	Positive rate	11390	108	0.95	0.588

Note: ILI% means the proportion of cases of influenza-like illness to the total number of outpatient visits. The positive rate means the rate of influenza virus isolation. Excess hospitalizations refer to the excess number of hospitalizations associated with ILI or influenza. The percentage of excess hospitalizations refers to the proportion of excess hospitalizations for ILI or influenza to the total number of hospital admissions.

* ILI% and positive rate represent the ILI activity and laboratory-confirmed influenza activity in the model, respectively.

### Impact of ILI and influenza on deaths

A total of 1019 patients died of respiratory disease in 2007–2008, of whom 177 died of ILI-associated respiratory diseases; that is, among the deaths due to respiratory diseases, 17.4% were ILI related. The impact of ILI on COPD was most significant (P < 0.05), with 33.1% of COPD-related deaths being due to ILI (Table 5).

**Table 5:** Impact of influenza-like illness on deaths by disease, sex, and age.

		No. deaths	No. excess deaths	Percentage of excess deaths (%)	P value
Cause of death	All respiratory disease	1019	177	17.4	0.23
	COPD*	472	156	33.1	0.03
	Others	547	28	5.1	0.82
Sex	Male	610	88	14.4	0.3
	Female	409	127	31.1	0.1
Age	elders(≥65 years)	884	181	20.5	0.17
	youngers(<65years)	142	40	28.2	0.4

Note:

* COPD: chronic obstructive pulmonary disease.

ILI had no significant effect on the number of deaths from respiratory diseases among populations by sex and age (P > 0.05); however, it was found that ILI seems to have a greater impact on women. Of the deaths due to respiratory diseases, 14% were ILI related for males and 31% for females.

By using the same methods to analyze the impact of influenza on the number of deaths, the constructed core model was used for the analysis of the influenza virus isolation rate. The obtained excess deaths were mostly negative, and the P values of the introduced influenza variable in the model were much higher than the 0.05 cutoff (average 0.8). The impact of influenza on the number of deaths requires further evaluation.

## Discussion

Zhuhai City has a typical subtropical climate, with hot and rainy summers and mild winters. Like in other subtropical cities (e.g., Hong Kong), influenza activity is seen throughout the year in Zhuhai City. Several epidemics with fluctuating intensity and seasonal peaks are seen throughout the year, making the regional prevention and control of influenza more complex [23, 24]. Influenza monitoring in Zhuhai City during 2007–2009 showed year-round local pathogen activity; however, different subtypes of influenza virus showed inconsistent epidemic peaks, suggesting that the active period of transmission of a specific influenza virus should be considered in the timing of influenza vaccination to ensure the best preventive effect.

Our study found an abrupt increase of influenza activity in 2009 compared with that in 2007 and 2008, and we believe that, in addition to the most obvious cause—the emergence of the new pandemic H1N1 strain—changes in surveillance practice (i.e., increased health-care-seeking behavior for mild illnesses during the pandemic period and expansion of the surveillance range) are another reason for the substantial increase in influenza detection in 2009.

Influenza surveillance includes epidemiological (i.e., ILI surveillance) and virological surveillance. Virological surveillance is theoretically the most powerful reflection of influenza activity. Owing to cost and other aspects, it is impossible to perform virological detection for all ILIs. During 2007–2009, only 2.8% of patients with ILI in Zhuhai City sentinel hospitals underwent influenza virological detection. Influenza is a self-limiting disease with nonspecific clinical symptoms, and a lack of laboratory testing would make it clinically difficult to distinguish influenza from other diseases with flu-like symptoms [25]. Fortunately, our data analysis found a positive correlation between the number of ILI cases and the influenza virus isolation rate by week during 2007–2009, and the strong correlation between ILI and virus activity was even more significant in the 2009–2010 pandemic year, indicating that ILI monitoring may reflect the influenza epidemic.

In this study, we found that ILI has a very significant impact on COPD. We determined that 32% of deaths due to COPD were attributable to ILI, indicating that patients with COPD need effective ILI prevention and control to reduce the risk of death, and that emphasis should be given to providing influenza vaccination for this part of the population. According to Wong et al., the impact of influenza on pneumonia and influenza (P&I) was more significant in Hong Kong, Guangzhou, and Singapore, and that influenza virus activity was highly correlated with P&I [26]. However, owing to missing data in this aspect, a related study in Zhuhai City has not been performed.

Influenza can be a direct cause of death, lead to secondary infections, or induce or cause deterioration of other diseases (e.g., congestive heart failure, COPD) that can result in death. During 2006–2008, 171 deaths were attributed to pneumonia in Zhuhai City, of which only 1 death was attributed to influenza. The mortality rate recorded in the death database is likely lower than the real situation for the following reasons: first, only a small number of patients with suspected influenza infections (especially adult patients) underwent virological testing; additionally, even in patients who were evaluated etiologically and ultimately died, the death certificates rarely listed influenza as the underlying cause of death owing to the doctors’ self-protection and other reasons [27]. The United States Centers for Disease Control and other researchers have stated that influenza mortality reflects a certain influence on patients who died of influenza but seriously underestimates the disease burden and that an appropriate statistical model is needed to estimate the full impact of influenza. The influenza-associated mortality estimated with the statistical model was 5–60 times that of the recorded influenza mortality rate in the United States [28–30]. A cohort study in Sydney, Australia, reported that patients with a discharge diagnosis of influenza only constituted one-sixth of all influenza-related hospitalizations [31].

In this study, the GAM was used to assess the impact of influenza and ILI on outpatient cases, hospitalizations, and deaths in the Zhuhai City community. The 2009 WHO Influenza Burden Study Guide stated that the method was particularly suitable for the year-round assessment of influenza activity in tropical and subtropical regions. The results showed that ILI had a substantial effect on the number of outpatient visits, hospitalizations, and deaths. Four percent of outpatient visits were attributable to ILI, whereas 7% of hospitalized patients’ diseases were associated with ILI and 17% of respiratory disease deaths were related to ILI. In addition to the influenza virus, many respiratory viruses (such as respiratory syncytial virus and adenovirus) and bacteria can cause ILI [29, 31], the epidemic seasons of which often overlap with those of influenza. Thus, the burden of ILI should include influenza and other related diseases involving respiratory pathogens and those with a much greater impact than the disease burden of influenza.

Here, we tried to use the positive rate of influenza virus detection as an indicator of influenza activity, to assess the strength of the influenza disease burden; however, we found that only the impact of influenza on outpatient attendance was statistically significant (nearly 2% of outpatients’ conditions were attributable to influenza virus infection). There is a need to further strengthen the virological surveillance of influenza in the future by increasing ILI virological detection and emphasizing the randomness of the sampling to avoid artificially increasing the virus isolation rate by deliberately collecting typical cases for influenza virus separation. Such selective bias will result in influenza virological surveillance not accurately reflecting the strength of the influenza epidemic.

In this study, only 157 weeks of data were available for 2007–2009 (the study of death involved only 105 weeks of data) and analyzed. This period is too short for a study on the influenza disease burden, which resulted in an inadequate test performance. Thus, further studies with more accumulated data are necessary. In this study, we only analyzed existing respiratory mortality data, and several similar studies found that influenza not only affects respiratory diseases but also induces or aggravates the risk for cardiovascular and other diseases. Therefore, it is necessary to analyze the data of all causes of deaths to enable a comprehensive assessment of the disease burden of influenza. Similarly, if detailed data of hospitalization cases (including patient age and sex, disease etiology, and other information) can be obtained, the disease burden of influenza can be analyzed and high-risk populations can be further identified.

## Supporting Information

S1 Fig Time series of deaths due to respiratory diseases in three hospitals of Zhuhai City in China, by week, 2006–2008. (TIF)

S1 Table Influenza virus isolations during 2007–2009, Zhuhai City, Guangdong Province, China. (DOCX)
