# Supplementary figures and images for: Impact of Influenza on Outpatient Visits, Hospitalizations, and Deaths by Using a Time Series Poisson Generalized Additive Model
Source: PLoS One. 2016 Feb 19;11(2):e0149468. doi: 10.1371/journal.pone.0149468 (PMC4760679; doi:10.1371/journal.pone.0149468)

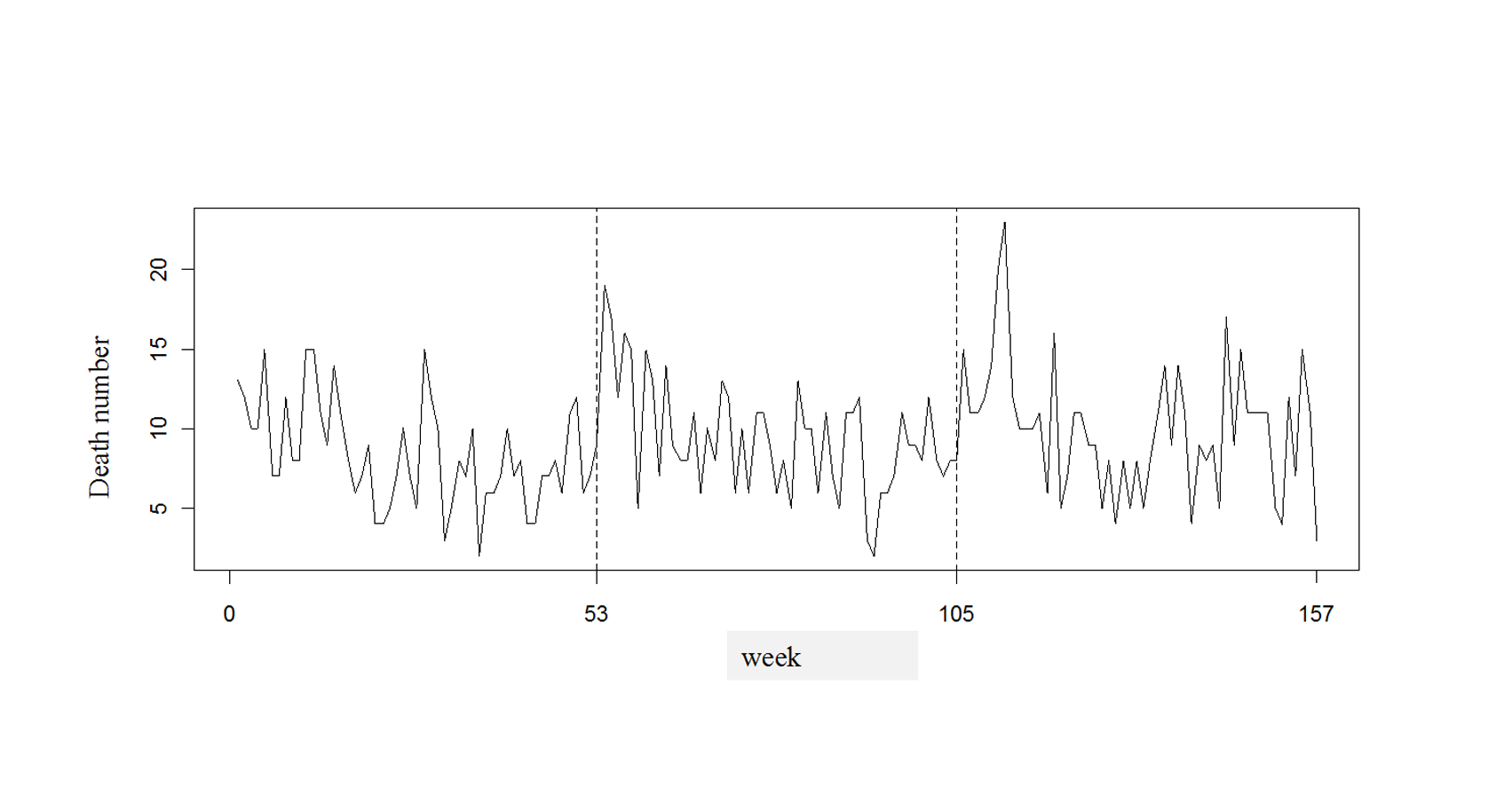

Supplement: S1 Fig — (TIF) [file pone.0149468.s001.tif]
