# Supplementary material for: Impact of Influenza on Outpatient Visits, Hospitalizations, and Deaths by Using a Time Series Poisson Generalized Additive Model
Source: PLoS One. 2016 Feb 19;11(2):e0149468. doi: 10.1371/journal.pone.0149468 (PMC4760679; doi:10.1371/journal.pone.0149468)
Supplement: S1 Table — (DOCX) [file pone.0149468.s002.docx]

**S1 Table Influenza virus isolations during 2007**–**2009, Zhuhai City, Guangdong Province of China.**

| Year | H1N1 | | H3N2 | | B | | Pandemic A(H1N1) | | Un-typed | | Total | |
| --- | --- | --- | --- | --- | --- | --- | --- | --- | --- | --- | --- | --- |
|  | No. isolations | Proportion (%) | No. isolations | Proportion (%) | No. isolations | Proportion (%) | No. isolations | Proportion (%) | No. isolations | Proportion (%) | No. isolations | Proportion (%) |
| 2007 | 0 | 0 | 50 | 79.4 | 8 | 12.7 | 0 | 0 | 5 | 7.9 | 63 | 7.9 |
| 2008 | 62 | 45.9 | 37 | 27.4 | 32 | 23.7 | 0 | 0 | 4 | 3 | 135 | 16.8 |
| 2009 | 56 | 9.3 | 62 | 10.3 | 55 | 9.1 | 358 | 59.3 | 73 | 12.1 | 604 | 75.3 |
| Total | 118 | 14.7 | 149 | 18.6 | 95 | 11.8 | 358 | 44.6 | 82 | 10.2 | 802 | 100 |
